# Alcoholic Extract of* Eclipta alba* Shows* In Vitro* Antioxidant and Anticancer Activity without Exhibiting Toxicological Effects

**DOI:** 10.1155/2017/9094641

**Published:** 2017-01-31

**Authors:** Navneet Kumar Yadav, Rakesh Kumar Arya, Kapil Dev, Chetan Sharma, Zakir Hossain, Sanjeev Meena, K. R. Arya, J. R. Gayen, Dipak Datta, R. K. Singh

**Affiliations:** ^1^Toxicology Division, CSIR-Central Drug Research Institute, Lucknow 226031, India; ^2^Biochemistry Division, CSIR-Central Drug Research Institute, Lucknow 226031, India; ^3^Botany Division, CSIR-Central Drug Research Institute, Lucknow 226031, India; ^4^Pharmacokinetics and Metabolism Division, CSIR-Central Drug Research Institute, Lucknow 226031, India; ^5^Academy of Scientific and Innovative Research (AcSIR), Anusandhan Bhawan, New Delhi 110001, India

## Abstract

As per WHO estimates, 80% of people around the world use medicinal plants for the cure and prevention of various diseases including cancer owing to their easy availability and cost effectiveness.* Eclipta alba* has long been used in Ayurveda to treat liver diseases, eye ailments, and hair related disorders. The promising medicinal value of* E. alba* prompted us to study the antioxidant, nontoxic, and anticancer potential of its alcoholic extract. In the current study, we evaluated the* in vitro* cytotoxic and antioxidant effect of the alcoholic extract of* Eclipta alba* (AEEA) in multiple cancer cell lines along with control. We have also evaluated its effect on different* in vivo* toxicity parameters. Here, we found that AEEA was found to be most active in most of the cancer cell lines but it significantly induced apoptosis in human breast cancer cell lines by disrupting mitochondrial membrane potential and DNA damage. Moreover, AEEA treatment inhibited migration in both MCF 7 and MDA-MB-231 cells in a dose dependent manner. Further, AEEA possesses robust* in vitro* antioxidant activity along with high total phenolic and flavonoid contents. In summary, our results indicate that* Eclipta alba* has enormous potential in complementary and alternative medicine for the treatment of cancer.

## 1. Introduction

Cancer is one of the most frequently occurring diseases causing 8.2 million deaths in 2012 worldwide. Further breast cancer is the most often diagnosed cancer in females and the leading cause of cancer related death worldwide, with an estimated 1.7 million new cases and 522,000 deaths around the world in 2012. There has been a great advancement in cancer treatment which enhanced the patient survival and quality of life. However, cancer related deaths are continuously rising [1–3].

Medicinal plants exhibit a wide range of biological properties for combating human diseases. A large number of medicinal plants were utilized all over the world for prevention and treatments of diseases [4]. Use of herbal medicine for the treatment of breast cancer and other types of cancer has been well reported in scientific literature [5–9]. Moreover, plant based medicines also have an enormous potential to provide low cost, easily accessible, and safe method of treatment [10, 11]. Herbal medicines are widely accepted in complementary and alternative medicine especially in cancer patients with poor socioeconomic condition. Hundreds of plants possessing anticancer properties have been identified, and they are the source of alternative medicine for cancer therapy in various regions of the globe [12–14]. However, a large number of plant species remain to be screened for their therapeutic potential; therefore, they can be used as a continuous source of new medicines for present and future health problems of humans including cancer.

Medicinal plants are also playing an important role in drug discovery and have been exploited for effective and beneficial uses against cancer, as both preventive and therapeutic strategies [13]. Generally prescribed chemotherapy drugs show very narrow therapeutic indexes and are known to cause severe side effects involving nausea, loss of taste, fatigue, loss of hair, immunosuppression, loss of libido, and myelosuppression, whereas herbal medicines are expected to have lesser undesirable effects in comparison to synthetic compounds and chemotherapeutic drugs [4–7, 15]. Herbal medicines provide us with relatively safe, effective, and economical therapeutic options, especially in the context of cancer in developing countries [16]. The nontoxic mode of antitumor activity of medicinal plants has been associated with their ability to selectively trigger multiple cell death pathways and thus induced apoptosis in cancer cells, sparing normal cells alive [17, 18].

*Eclipta alba* (L.) Hassk (synonym* Eclipta prostrata*) is an annual herbaceous plant, erect or prostrate, belonging to the Asteraceae family. It is also known as Bhringaraj in Ayurveda which has been generally utilized for a very long time as a part of conventional prescription for ailments especially related to the liver and hair. There are four main varieties of the herb* Eclipta alba* based on the colour of their blossom, that is, red, yellow, white, and blue. The white and yellow ones assume an essential part in traditional medicine, but it is the white species (*Eclipta alba*) that is most commonly harvested for its therapeutic advantages as it grows wildly in moist places, as a weed, and it can be easily propagated. The extracts from the leaves and flowers of this medicinal herb can be applied in numerous ways, both topically and internally, to soothe many ailments.

In the Ayurvedic system of medicine,* Eclipta alba* was used to maintain the balance of Vata and Kapha and described as medicinal herb for the treatment of liver diseases [19]. In China, it has been traditionally used as a food additive and herbal medicine to cure infectious hepatitis, liver cirrhosis, abnormal uterine bleeding, jaundice, hematuria and diarrhea with bloody stools, epistaxis, aching and weakness of the knees and loins, and spitting of blood [20–23].* Eclipta alba* shows versatile pharmacological effects that include hair growth, antimicrobial, antioxidant, anti-inflammatory, analgesic, antinociceptive, antileprotic, antihaemorrhagic, antimyotoxic, antiviral, antihepatotoxic, diuretic, hypotensive, hypocholesterolemic, hypotensive, immunomodulatory, nootropic, ovicidal, and spasmogenic activity [24–34].

Hepatoprotective activity of* Eclipta alba* is well described in literature [33, 35–38]. In Indian systems of medicines herbal decoction of* Eclipta alba* is used for the treatment of liver cirrhosis, infective hepatitis (liver inflammation due to a viral infection), liver enlargement, and other related diseases of liver and gall bladder [39–42]. Various scientific studies reported that* Eclipta alba* shows protective effects in rats and mice against CCl_4_-induced liver damage. Its protective activity against liver injury and inflammation produced the effects on subcellular levels [19, 36, 39].* Eclipta alba* have been traditionally used as a hair growth promoter and several scientific studies also confirmed this potential [25, 33, 34, 43, 44].* Eclipta alba* acts as an important exogenous mediator that stimulates follicular keratinocyte proliferation and delays terminal differentiation by downregulating TGF-*β*1 expression. Thus it can be used in treatment of certain types of alopecia [45].

Lirdprapamongkol et al., 2008, first reported the inhibitory effects of* Eclipta alba* on invasion, migration, and adhesion of cancer cells. Further, they also determined the antiangiogenic activity by using chick chorioallantoic membrane assay [46]. Chaudhary et al., 2011, showed cytotoxic effects of* Eclipta alba* on HepG2 (human liver), A498 (rat kidney), and C6 (rat glioma) cell lines [47]. They also reported* in vitro* and* in vivo* multidrug resistance reversal potential of* Eclipta alba* on hepatocellular carcinoma [48, 49]. Chauhan et al., 2012, and Liu et al., 2012, described its anticancer activity on human hepatocarcinoma cell line (SMMC-7721) and human lung epithelial adenocarcinoma cell line (HCC-827), respectively [22, 32]. Ali et al., 2014, reported its chemopreventive effects on skin carcinogenesis [50]. Further, Kim et al., 2015, and Cho et al., 2016, showed the growth inhibitory effects of* Eclipta alba* constituents on ovarian cancer cells [51, 52].

Reactive oxygen species (ROS) plays a critical role in inducing antitumor effects in most of the herbal medications. At the optimum level, ROS acts as a prosurvival factor, whereas high level of ROS is responsible for irreversible damage to DNA, protein, and lipids, resulting in cell death through various ways, including necrosis and apoptosis [53–55]. In the case of stress, ROS increased beyond its normal level causing serious injury to cellular system and subsequently gave rise to many diseases such as diabetes, atherosclerosis, inflammation, aging, cardiovascular diseases, and cancer [56]. Thus, equilibrium in ROS level is very important for normal body function. Numbers of phytocompounds present in medicinal plant extracts have potent antioxidant property. Multiple reports have described the ROS scavenging activity of various phytocompounds and plant extract. Hence, they can be used for protective activity against ROS mediated damage [57–59].

Although anticancer properties of* Eclipta alba* extract have recently been reported, here, we have reported the anti-breast cancer activity of alcoholic extract of* Eclipta alba* (AEEA) along with its antioxidant activity and* in vivo* toxicity study. Results of anticancer and antioxidant study will be crucial for understanding the role of AEEA in breast cancer therapy.* In vivo* study was conducted to evaluate the safety of AEEA administrated orally. Outcomes of this study will be helpful in advancement of knowledge regarding any unwanted side effects associated with use of AEEA in therapy.

## 2. Materials and Methods

### 2.1. Ethics Statement

All animal procedures have been approved and prior permission from the Institutional Animal Ethical Committee (IAEC) was obtained as per the prescribed guidelines.

### 2.2. Reagents

Hoechst 33342 and Rhodamine 123 were obtained from Sigma Aldrich. Annexin V Alexa Fluor 488 was procured from Molecular Probes-Invitrogen. All chemicals and antibodies were obtained from Sigma unless specified otherwise.

### 2.3. Plant Material

*Eclipta alba* was collected and the sample was authenticated by Dr. K. R. Arya, Principal Scientist, Botany Division, CSIR-Central Drug Research Institute Lucknow (UP), India. Specimen sample of* Eclipta alba* has been allotted a voucher sample specimen number KRA/2275 and kept at the medicinal plant repository of the institute.

### 2.4. Preparation of* Eclipta alba* Extract

The* Eclipta alba* was dried in an oven at 40°C for 5 days and then ground in an electric blender. The powder was suspended in 80% alcohol and left at room temperature for 24 h. The crude extract was filtered using 125 mm Whatman® qualitative filter paper under sterile condition. This process was repeated 5 times and then the solvent (alcoholic extract of* Eclipta alba*), thus collected, was evaporated to dryness under reduced pressure using a rotary evaporator below 50°C. Residues were further subjected to dryness by incubating them for 8 days at 37°C and extract was kept at 4°C until use. The yield of the extract was 12.5% (w/w).

### 2.5. Quantification of Total Phenol and Total Flavonoid

Total phenol content of the samples was determined by Folin-Ciocalteu's calorimetric method as described by Singleton and Rossi, 1965 [60]. Folin-Ciocalteu reagent reduced the samples containing polyphenols producing blue coloured complex. Quantification was carried out on the basis of the standard curve of gallic acid prepared in 80% (v/v) methanol. To prepare a calibration curve, 100 *μ*L aliquots of 12.5, 25, 50, 100, 200, and 400 *μ*g/mL methanolic gallic acid solutions were mixed with 250 *μ*L Folin-Ciocalteu reagent (diluted tenfold) and 250 *μ*L (7.5%) sodium bicarbonate. After incubation at 25°C for 30 min, absorbance was taken at 765 nm against blank by UV spectrophotometer. The same procedure was applied for the samples as described above in the preparation of calibration curve.

Total flavonoid content (TFC) was determined using aluminium chloride colourimetric method described by Chang et al., 2002 [61]. Results were expressed as mg quercetin equivalent/g dry weight (DW). The calibration curve was prepared using standard quercetin compound at concentrations of (12.5–400 *μ*g/mL) in methanol. 0.5 mL of each plant extract in methanol was separately mixed with 1.5 mL of methanol, 0.1 mL of 10% aluminium chloride, 0.1 mL of 1 M potassium acetate, and 2.8 mL of distilled water. After the incubation of the reaction mixture at room temperature for 30 min, the absorbance was determined using spectrophotometer at *λ*_max_ = 415 nm against the blank solution. All measurements were performed in triplicate and results of TPC and TFC were expressed in mg gallic acid equivalent (GAE) and mg quercetin equivalent (QUE) per gram of dry weight of sample, respectively.

### 2.6. *In Vitro* Antioxidant Activity and Free Radical Scavenging Capacity

Stock solution of each sample was prepared in a concentration of 1 mg/mL. Standard (i.e., ascorbic acid and quercetin) was used for comparison in all assays. The absorbance at the respective wavelengths was recorded using a UV-vis spectrophotometer and the measurements were run in triplicate.

### 2.7. Total Antioxidant Capacity

Total antioxidant activity of the AEEA was evaluated by the phosphomolybdate assay (Prieto et al., 1999), with slight modifications [62]. An aliquot of 0.5 mL of extract solution was added to 5 mL of reagent solution (0.6 M sulfuric acid, 28 mM sodium phosphate, and 4 mM ammonium molybdate). The reaction solutions were allowed to incubate in boiling water bath at 90°C for 90 min. After cooling the samples at room temperature, the absorbance of the solution was measured at 695 nm against blank using a spectrophotometer. The same method was used for blank sample and 0.5 mL methanol is used instead of extract. Total antioxidant capacity was calculated using the standard graph of ascorbic acid. Results are expressed as equivalent of ascorbic acid in mg per gram of extract.

### 2.8. DPPH Radical Scavenging Activity

The antioxidant activity of the AEEA was measured in terms of hydrogen donating ability using the traditional DPPH assay of Brand-Williams et al., 1995, with slight modifications [63]. 0.5 mL of each sample and control at various concentrations (3.125–100 *μ*g/mL) was added to 1.5 mL DPPH (0.004%) solution and allowed to stand in the dark at room temperature for 20 min. The absorbance at 517 nm was recorded using a UV-vis spectrophotometer and results are expressed in mM ascorbic acid equivalent (AAE) per g dry weight of sample. The percentage of inhibition of DPPH free radical scavenging activity was calculated using the following equation:

(1) $$\begin{array}{c} \%\;\;inhibition=\left[\;\frac{\left(A_{DPPH}-A_{sample}\right)}{A_{DPPH}}\;\right]\times 100, \end{array}$$

where *A*_DPPH_ is absorbance of DPPH and *A*_sample_ is absorbance of sample (extract/ascorbic acid).

### 2.9. Nitric Oxide Free Radical Scavenging Activity

To measure the nitric oxide free radical scavenging activity, 0.1 mL of plant extract of different concentration dissolved in DMSO was taken and then methanol was added to make the volume 150 *μ*L. 2.0 mL of sodium nitroprusside (10 mM) in phosphate buffer saline was added in each tube and all samples were incubated at room temperature for 150 minutes. After the incubation, 5 mL of Griess reagent was added to each tube and the absorbance of chromophore formed was measured at 546 nm on spectrophotometer. Same procedure was repeated with standard ascorbic acid and methanol (blank which served as control) [64, 65]. The percentage scavenging activity was calculated using the following equation:

(2) $$\begin{array}{c} \%\;\;Scavenging=\left[\;\frac{A_{control}-A_{sample}}{A_{control}}\;\right]\times 100, \end{array}$$

where *A*_control_ is absorbance of control and *A*_sample_ is absorbance of sample (extract/ascorbic acid).

### 2.10. Lipid Peroxidation Inhibition Activity

MDA assay was used to determine the lipid peroxidation inhibition effect of* E. alba* extract (AEEA) as described by Baharum et al., 2014. Briefly, rat liver tissue (2 gm) was sliced and homogenized with 10 mL KCl-Tris-HCl buffer (pH 7.2). The reaction solution (0.5 mL liver homogenate, 0.2 mL Tris-HCl buffer (pH 7.2), 0.1 mL ascorbic acid, and 0.1 mL 4 mM FeCl_2_) and 0.1 mL of plant extract were taken in tube. The reaction tube was incubated at 37°C for 1 h. After incubation 1 mL 1N HCl, 0.4 mL 9.8% sodium dodecyl sulfate (SDS), 1.8 mL distilled water, and 4 mL 0.6% TBA were added to each tube and vigorously shaken. Then, the tubes were placed in a boiling water bath at 100°C for 30 min. After cooling, the flocculent precipitate was removed by adding 5 mL n-butanol, mixed well, and centrifuged at 9000 rpm for 10 min. The absorbance of the supernatant was measured at 532 nm [66]. The percentage of lipid peroxidation was measured using following equation:

(3) Lipid  peroxidation  inhibition  %=Acontrol−AsampleAcontrol×100,

where *A*_control_ is absorbance of control and *A*_sample_ is absorbance of sample (extract/ascorbic acid).

### 2.11. Measurement of Reactive Oxygen Species (ROS)

Intracellular ROS level was determined by using peroxide-sensitive fluorophore 2′,7′-dichlorodihydrofluorescein diacetate (DCFH_2_-DA) (Sigma). HEK 293 cells were seeded in black bottom 96-well plate and grown up to the confluence. Cells were now treated either with or without different concentrations of AEEA for 24 h. Following treated cells were briefly washed with PBS and incubated with DCFH_2_-DA 10 *μ*g/mL for 30 minutes at 37°C under atmosphere pressure, including 5% CO_2_. Cells were again washed twice with PBS and fluorescence absorbance was measured at 495/525 nm. The reading was taken on fluorimeter (POLARstar Galaxy; BMG Labtech, Mount Eliza, VIC, Australia).

### 2.12. Test Animals

CF rats (150–175 gm) were obtained from the National Laboratory Animal Center (NLAC), Central Drug Research Institute, Lucknow, India. The animals were housed in polycarbonate cages with bedding at 25 ± 2°C temperature and 30–60% relative humidity with a 12 h light and dark cycle throughout the study period. CF rats were allowed to acclimatize to experimental room conditions for 7 days prior to toxicity study. The animals were fed a standard rodent pellet diet and water* ad libitum* [67–71].

### 2.13. Toxicity Study

Healthy CF rats were randomly divided into five groups, with 5 animals per group. One group served as the control and received 1% gum acacia in distilled water. Four other groups were orally treated by gavage with different doses of AEEA (500, 1000, 1500, and 2000 mg/kg) suspended in water with 1% gum acacia. A toxicity study was carried out as recommended by toxicity evaluation guideline of Schedule Y [72].

Rats were observed for toxicity symptoms as defined by the Common Toxicity Criteria developed by the Cancer Therapy Evaluation Program with some modification if needed (National Cancer Institute, 1999, Common Toxicity Criteria Version 2.0, Cancer Therapy Evaluation Program). Their body weight changes and food and water intake were recorded on alternate days.

At the end of the study, the animals were fasted overnight, although water was made available* ad libitum*. They were then anesthetized using diethyl ether for necropsy and blood collection. Blood was collected in two different tubes: one tube containing the anticoagulant EDTA and one tube without anticoagulant for hematological and biochemical examination, respectively. The vital organs of animals were dissected and removed with care. Weight of each organ was taken and examined for macroscopic features.

### 2.14. Hematological and Biochemical Analysis of Blood

Blood collected in EDTA coated vials was analyzed using MS-9 automatic hematology analyzer (Melet Schloesing Ltd., France), shortly after its collection. Blood samples were collected for serum chemistry analysis in tubes lacking anticoagulant and placed at room temperature for at least 90 min prior to centrifugation; after centrifugation at 1600*g* for 10 min., serum was collected and biochemical parameters were measured using fully automated random access clinical chemistry analyzer (Beckman Synchron CX5, USA).

### 2.15. Cell Culture

MCF 7, 4T1, MDA-MB-231, HeLa, SK-OV-3, SW620, DU145, A549, PANC-1, VERO, and HEK-293 cells were obtained from the American Type Culture Collection (ATCC), resuscitated from early passage liquid nitrogen vapor stocks as needed, and cultured according to the supplier's instructions. Cells were routinely inspected microscopically for stable phenotype.

### 2.16. Cytotoxicity Assay

A standard colourimetric SRB (sulforhodamine B) assay was used for the measurement of cell viability as described before. Briefly, 10,000–20,000 cells (depending on the doubling time of each cell type) were seeded to each well of 96-well plate in 5% serum containing growth medium and incubated overnight in CO_2_ incubator at 37°C. Adhered cells were then treated with vehicle or AEEA at the required dose. After 48 h of exposure, cells were fixed with ice-cold 50% TCA, stained with 0.4% (w/v) SRB in 1% acetic acid, washed, and air dried. Bound dye was dissolved in 10 mM Tris base and absorbance was measured at 510 nm on a plate reader (Epoch Microplate Reader, Biotek, USA). The cytotoxic effects of the compound were calculated as % of cell viability as per the formula 100 − [100 − (absorbance of treated cells/absorbance of vehicle treated cells)] × 100.

### 2.17. Detection of Apoptosis

Externalization of phosphatidylserine in apoptotic cells was detected by Annexin V FITC, (green-fluorescent dye). In brief, MCF 7 and MDA-MB-231 cells were grown on coverslips for 24 h and then treated with different concentration of AEEA for 24 h. Control and treated breast cancer cells were stained with Annexin V FITC and analyzed under fluorescent microscope.

### 2.18. Microscopic Analysis by Hoechst Staining

Morphological changes in the nucleus induced by AEEA treatment were studied by Hoechst 33342 staining. MCF 7 cells (2 × 10^4^/well) were seeded in 24-well plate and after 24 h of growth cells were treated with different concentration of AEEA for 24 h and cells were fixed with 4% paraformaldehyde for 10 min and then washed with PBS and permeabilized with 4% paraformaldehyde containing 0.5% triton X-100 for 30 min and then stained with Hoechst 33342 stain (Invitrogen 3 mg/mL) for 30 min and images were captured by microscope (Leica).

### 2.19. Determination of Mitochondrial Membrane Potential (ΔΨ*m*)

The changes in the mitochondrial potential were detected by Rhodamine 123, a cationic dye that exhibits potential dependent accumulation in mitochondria, indicated by fluorescence emission shift [73]. In brief, MCF 7 cells grown on coverslips were treated with different concentration of AEEA for 24 h. Control and treated breast cancer cells were stained with Rhodamine 123 and analyzed under fluorescent microscope.

### 2.20. Wound Healing Assay or Migration Assays

MCF 7 and MDA-MB-231 cells were plated in 6-well plates. Upon reaching 70–80% confluency, the scratch was performed scraped with a p200 pipette tip. Once the scratch was made the media were removed and replaced with fully supplemented media containing different concentrations of AEEA. Images of the scratch were taken under microscope immediately after the scratch was induced (0 h) and after 24 h.

### 2.21. Chemical Analysis by Mass Spectrometry

For chemical characterization mass spectrometric detection was performed on API 4000 Q TRAP mass spectrometer (AB SCIEX Toronto, Canada) equipped with an electrospray ionization (ESI) source. The AEEA was dissolved in 50 : 50 solution of A, 10 mM ammonium acetate, 0.1% formic acid in water, and B: 50 : 50 ACN : MeOH, and infused with Harvard Infusion Pump 11 (Harvard Apparatus, USA) with optimized flow rate of 20 *μ*L/minute.

The extract was scanned in both positive and negative ion mode within a range of 100 to 800 *m*/*z*, where the positive ion mode showed greater ionization and sensitivity. Data profiling was recorded at a speed of 0.15 s/scan and the scanning delay of 0.01 s during analysis. The main working parameters of the mass spectrometer were (i) ion spray voltage (ISV) 5500, (ii) curtain gas (CUR) 25, and (iii) ion source gases one (GS1) and two (GS2) 10 and quadruple set on unit resolution. Data processing was performed using Analyst version 1.5 software package (SCIEX).

### 2.22. Statistical Analysis

The data generated during the study was analyzed using one-way ANOVA test and the *p* value less than 0.05 was considered to be significant.

## 3. Results

### 3.1. Determination of Total Phenolic Content

**Figure 1:**
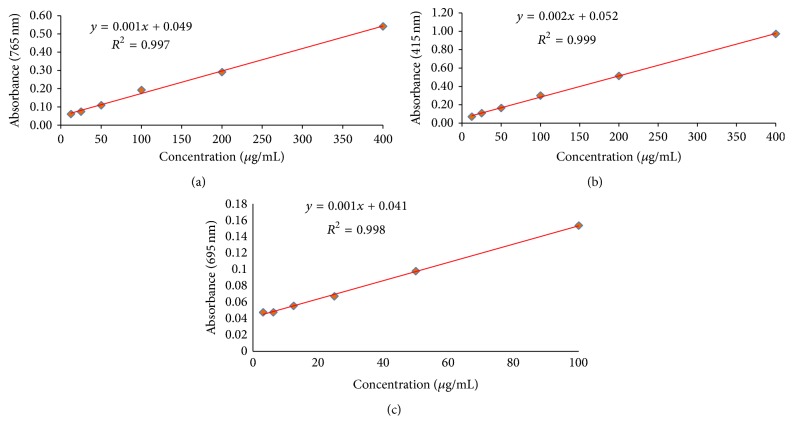
Calibration curve of standards: gallic acid (a), quercetin (b), and ascorbic acid (c) (each point (*R*^2^ values) represents the mean data set of *n* = 3).

Total phenolic content was estimated using a standard curve of gallic acid (*R*^2^ = 0.997; Figure 1(a)) and expressed as milligrams of gallic acid equivalent (GAE) per gm dry weight. The AEEA sample exhibited the TPC value 31.67 ± 3.82 mg of GAE/g of extract (Table 1).

### 3.2. Estimation of Total Flavonoid Contents

Total flavonoid contents (TFC) of the sample were determined using the standard curve of quercetin (*R*^2^ = 0.999; Figure 1(b)). The result was expressed in mg of QUE per gm of dry sample. The total flavonoid contents of AEEA sample were found to be 91.67 ± 5.05 mg of QUE/g DW (Table 1).

### 3.3. *In Vitro* Antioxidant Activity and Free Radical Scavenging Power

In order to evaluate the* in vitro* antioxidant potential of AEEA, various types of assay [i.e., total antioxidant activity (TAA), 1,1-diphenyl-2 picrylhydrazyl (DPPH) radical scavenging, lipid peroxidation inhibition activity (MDA assay), and nitric oxide (NO) free radical scavenging assay] were used in the current study.

### 3.4. Total Antioxidant Capacity

Total antioxidant activity (TAA) of the samples was obtained from the calibration curve of standard ascorbic acid as shown in Figure 1(c) and expressed as the number of equivalents of ascorbic acid (AAE). The AEEA sample showed the total antioxidant activity with a value 173.33 ± 5.77 mg equivalent of ascorbic acid (compared to standard ascorbic acid 726.57 ± 9.33 mg GAE per g DW) (Table 1).

### 3.5. DPPH Radical Scavenging Activity

The antioxidant scavenging capacity of plant extract (AEEA) was determined using the DPPH free radical scavenging assay. The antioxidant potential of the samples with the standard antioxidant ascorbic acid was evaluated using the parameter IC_50_. During the test, the degree of decolourization of DPPH from purple to yellow colour indicates the scavenging efficiency of the extracts [74]. The sample (AEEA) along with ascorbic acid revealed the highest scavenging activity of 25.62% and 63.75% at the concentration of 25 *μ*g/mL with IC_50_ value of 136.57 ± 6.83 and 33.5 ± 1.03 *μ*g/mL, respectively (Table 1). Therefore, it is reported that the smaller IC_50_ values show the higher antioxidant activity of the plant extracts [62], because the flavonoids and tannins are phenolic compounds and plant phenolics are a major group of compounds that serve as free radical scavengers [75]. However, the* in vitro* antioxidant capacity of extract (AEEA) was found to be lower (*p* < 0.05) than of standard ascorbic acid.

### 3.6. Nitric Oxide Scavenging Activity

Nitric oxide scavenging activity was performed against the sample (AEEA) along with the standard ascorbic acid. The sample (AEEA) along with ascorbic acid exhibited the maximal percentage inhibition (21.90% and 47.77%) at the concentration of 25 *μ*g/mL with IC_50_ value of 308.73 ± 6.34 and 66.27 ± 1.55 *μ*g/mL, respectively (Table 1).

### 3.7. Lipid Peroxidation Inhibition Activity

Lipid peroxidation inhibition activity was measured* in vitro* by determining the production of malondialdehyde (MDA) and related compounds in rat liver homogenate [76]. The lipid peroxidation occurs in nonpolar region of the biological membranes and produced the free radicals that lead to various human diseases including cancer [77]. Thus, the inhibition of lipid peroxidation is an indicator of therapeutic potential of plant extracts. The anti-lipid peroxidation activity of the sample along with standard ascorbic acid is illustrated in Table 1. The sample (AEEA) along with ascorbic acid showed the maximal percentage inhibition activity (19.58% and 36.36%) at the concentration of 25 *μ*g/mL with IC_50_ value of 126.99 ± 8.10 and 55.64 ± 3.27 *μ*g/mL, respectively (Table 1). The results suggest that the AEEA sample was found with a good anti-lipid peroxidation activity (i.e., inhibition of MDA formation) but less effective than ascorbic acid.

### 3.8. Effect of AEEA on Intracellular ROS Level

**Figure 2:**
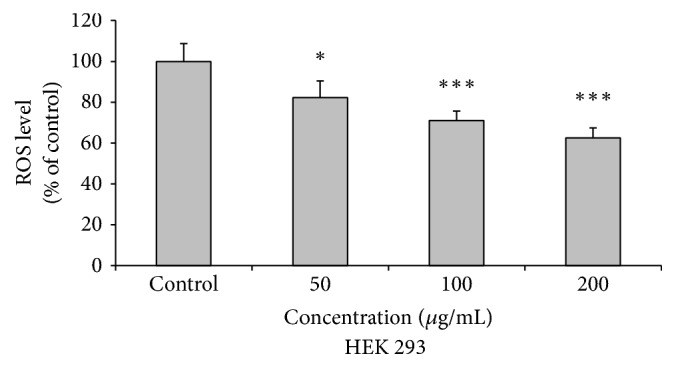
Intracellular ROS level of HEK 293 cells. Cells are treated with different concentration of AEEA for 24 h. Results are represented as mean ± SD. Statistical significance determined as compared to control (0.05 ≥ *p*). *∗* represents *p* ≤ 0.05; *∗∗∗* represents *p* ≤ 0.001.

ROS are produced inside the cells under various stress conditions. High level of ROS caused damage to cell and thus was responsible for occurrence of various diseases [78, 79]. Natural products which have potential to reduce the ROS level of normal cells were very useful in treatment and prevention of many diseases [80–83]. In the present study, we determined the intracellular ROS levels in HEK-293 cells by 2′,7′-dichlorodihydrofluorescein diacetate (DCFH_2_-DA). DCFH_2_-DA was hydrolyzed inside the cells by esterases to form nonfluorescent DCFH_2_, which subsequently are oxidized by intracellular ROS and produce fluorescence. Here, HEK-293 cells were treated with different concentrations of AEEA, and after 24 h ROS level was determined. Results of this study indicate that AEEA reduces the intracellular ROS levels in concentration dependent manner (Figure 2).

### 3.9. Antiproliferative Effect of AEEA on the Growth of Different Cancer Cell Lines

**Figure 3:**
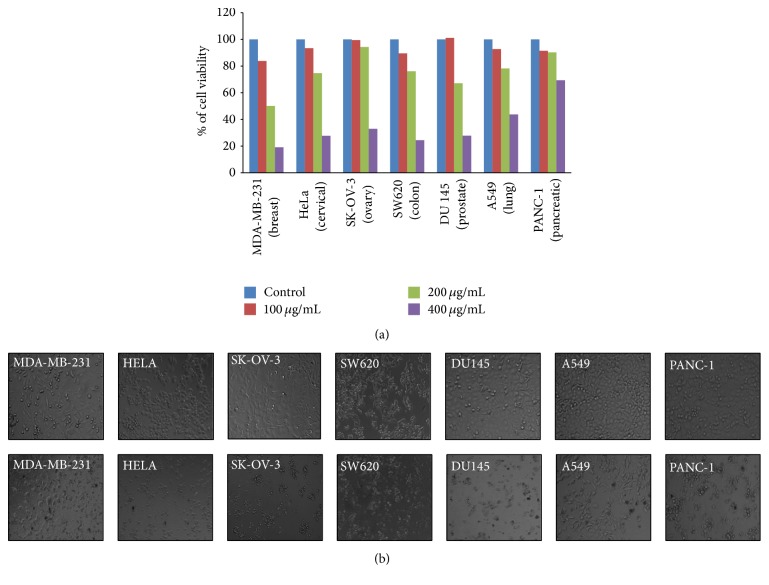
AEEA induced growth inhibition in different cancer cell lines. MDA-MB-231 (breast), HeLa (cervical), SK-OV-3 (ovary), SW620 (colon), DU145 (prostate), A549 (lung), and PANC-1 (pancreatic) were treated with different concentrations of AEEA for 48 h and cytotoxicity was measured as described in Materials and Methods. Percentage cell viability was plotted in graphs (a). Brightfield microscopy. Effects of AEEA on the morphological changes in different cancer cells after 24 h treatment (400 *μ*g/mL) were monitored by phase contrast microscopy (b).

**Figure 4:**
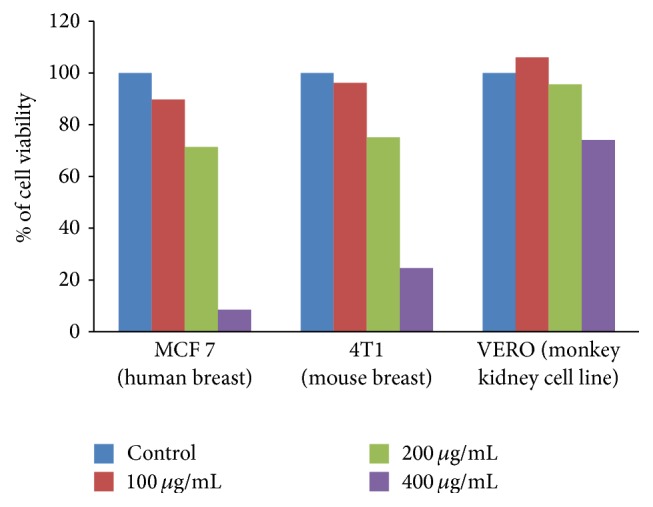
Cytotoxic effects of AEEA on human breast cancer cell line MCF 7, mouse breast cancer cell line 4T1, and normal epithelial cell line of African green monkey Vero. MCF 7, 4T1, and Vero cells were treated with different concentrations of AEEA for 48 h and cytotoxicity was measured as described in Materials and Methods.

In this study, we investigated the dose dependent* in vitro* cytotoxicity of AEEA on seven different cancer cell lines MDA-MB-231 (breast), HeLa (cervical), SK-OV-3 (ovary), SW620 (colon), DU145 (prostate), A549 (lung), and PANC-1 (pancreatic), belonging to seven different cancer types. Interestingly, we found AEEA to exhibit cytotoxic effect in all the cancer cell lines but it is most potent in inducing cytotoxic effects against breast cancer cells line (Figure 3(a)). Representative phase contrast microscopic images of vehicle or treated (400 *μ*g/mL of AEEA for 48 hr) cells of seven different cancers showed a clear morphological change. Cells were found to be shrink in size with ruffling and blebbing at cell membranes, suggesting the cells undergoing apoptosis (Figure 3(b)). Further, to test its selective cytotoxicity against cancer cells, we exploited the standard SRB assay to evaluate the cytotoxic effects of AEEA on human breast cancer cell line MCF 7, mouse breast cancer cell line 4T1, and normal epithelial cell line of African green monkey Vero. In our efficient efforts, we observed that AEEA is potently cytotoxic to breast cancer cells, but its toxicity is very minimal to nontumorigenic epithelial cells Vero, implying the nontoxic nature of AEEA (Figure 4).

### 3.10. Detection of Apoptosis Using the Annexin V FITC

**Figure 5:**
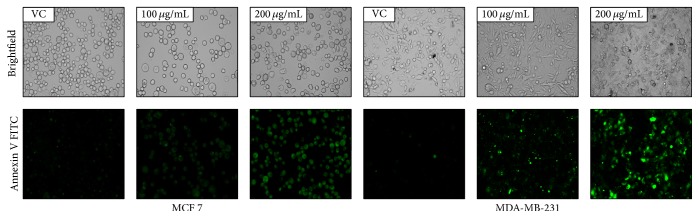
Detection of apoptosis in AEEA treated MDA-MB-231 using the Annexin V FITC. MCF 7 and MDA-MB-231 cells were treated with different concentrations of AEEA for 24 h and stained with Annexin V FITC following standard protocol and observed under fluorescence microscope.

One of the earliest signs of apoptosis is the loss of plasma membrane integrity. In the cell undergoing apoptosis, membrane phospholipid phosphatidylserine (PS) is translocated from the inner surface to outer surface of the plasma membrane [84]. Annexin V is a 35-36 kDa Ca^2+^-dependent phospholipid-binding protein with high affinity for PS, which can bind to exposed apoptotic cell surface PS. Annexin V can be conjugated to fluorochromes while retaining its high affinity for PS and thus serves as a sensitive probe for identification of early and late apoptotic cells. In present study MCF 7 and MDA-MB-231 were treated with different concentrations of AEEA for 24 h and then stained with Annexin V FITC for 30 min and observed under fluorescence microscope. Here we show that green fluorescence (Annexin V FITC) was increased dose dependently which represented that AEEA causes the translocation of phospholipid phosphatidylserine (PS) to the outer surface of the plasma membrane and thus is responsible for the onset of an early stage of apoptosis in both MCF 7 and MDA-MB-231 (Figure 5).

### 3.11. Hoechst 33342 Staining to Determine the Morphological Changes in Nucleus

**Figure 6:**
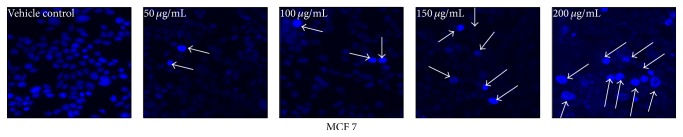
Detection of DNA damage by AEEA induced apoptosis in tumor cells by Hoechst 33342 staining. MCF 7 cells were seeded in 24-well culture plate and allowed to grow for 24 h. After treatment of 24 h with different concentrations of* Eclipta alba* extract cells were stained with Hoechst 33342 following standard protocol and observed under fluorescence microscopy.

Hoechst 33342 is membrane permeable blue fluorescent dye used for visualization of nuclear morphology changes and apoptotic body formation that are characteristics of apoptosis [85]. After staining with Hoechst 33342 stain, live cells are visualized with homogeneous light blue nuclei when observed under fluorescence microscope. Cells undergoing apoptosis display a bright blue colour after staining due to karyopyknosis and chromatin condensation [86, 87]. In present study Hoechst 33342 staining was performed to reveal the apoptosis inducing activity of AEEA in MCF 7 cell line. Results of this study confirmed the increase in fluorescence due to chromatin condensation in MCF 7 cells as compared to vehicle control in dose dependent manner (Figure 6). These results indicated that AEEA could induce apoptosis in breast cancer cell line.

### 3.12. Determination of Mitochondrial Membrane Potential (ΔΨ*m*)

**Figure 7:**
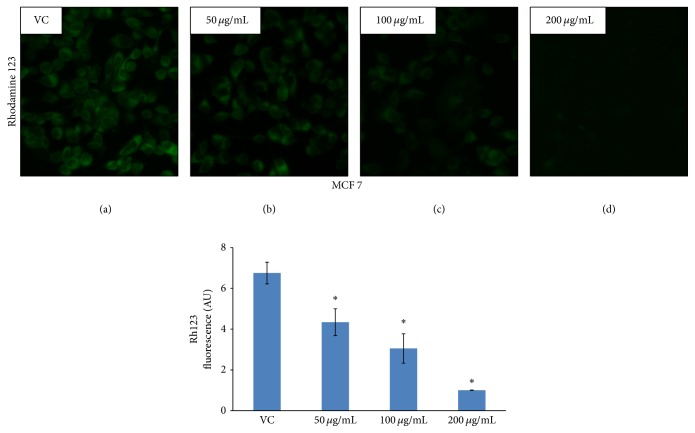
Determination of mitochondrial membrane potential (ΔΨ*m*). Rhodamine 123 (RH-123), a mitochondria specific fluorescent dye which stains live cells, is used to determine the mitochondrial membrane potential by measuring fluorescence intensity under microscope. MCF 7 cells were treated with different concentrations of AEEA for 24 h and stained with Rhodamine 123 following standard protocol and observed under fluorescence microscopy. (a) Control (without treatment); (b–d) treatment with various concentrations of* Eclipta alba* extract. *∗* represents *p* ≤ 0.05.

Loss in mitochondrial membrane potential is a crucial event of cell signaling pathway that leads to apoptosis. During the course of apoptosis, there is a decrease in mitochondrial membrane potential causing release of cytochrome *c* from mitochondrial intermembrane space. Release of cytochrome *c* thus initiated the downstream signaling of apoptosis. Rhodamine 123 is a fluorescent dye used to indicate the loss of mitochondrial membrane potential. In normal condition it permeabilized inside the mitochondria and emits green fluorescence. When decrease in mitochondrial membrane potential occurred, dye translocated outside the mitochondria. Hence, the loss of potential will result in loss of the dye and therefore the fluorescence intensity when viewed under microscope [88, 89]. After treatment of breast cancer cells with AEEA, the loss of fluorescence was observed in a dose dependent manner (Figure 7).

### 3.13. Wound Healing Assay or Migration Assays

**Figure 8:**
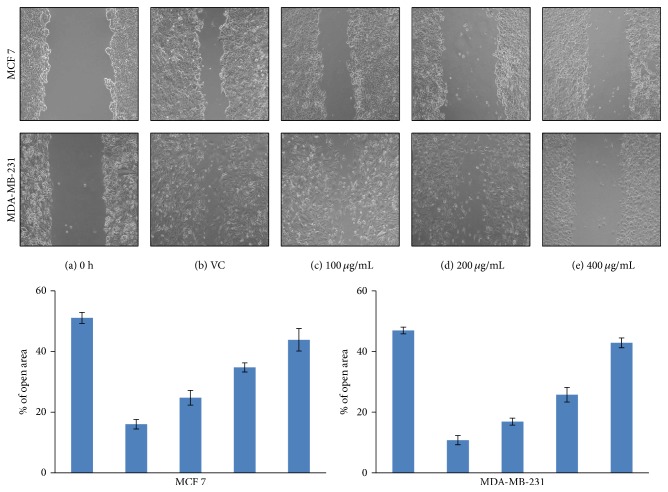
Cell migration inhibition by* Eclipta alba* extract. A scratch wound healing assay was performed on MCF 7 and MDA-MB-231 cells treated with different concentration of AEEA to determine the cell migration ability. (a) Showing scratch wounds in MDA-MD-231 cells at time 0. (b–e) Representing the status of wound at 24 h after the initiation of the scratch when the cells were treated with the vehicle control, DMSO (b), or different concentrations of AEEA (c–e). Wounds were created and AEEA was added immediately. Wounds were evaluated at 24 h after AEEA administration. ^*∗*^Indicating values that were significantly different (*p* < 0.05) from the DMSO control.

A wound healing assay was performed to observe the effect of AEEA on the migration ability of breast cancer cells. MCF 7 and MDA-MB-231 were treated with different concentrations of AEEA, and after 24 h cell migration status was determined under the microscope. Results of this study show that wound was almost healed in control group, but in treated groups inhibition was observed in both cells. In case of MCF 7 cells, migration of cells is inhibited at lower concentration of 100 *μ*g/mL and increases dose dependently. Concentration of 400 *μ*g/mL of AEEA almost completely inhibits the migrations of cells (Figure 8). Migration of MDA-MB-231 cells was also inhibited at a lower concentration of 100 *μ*g/mL. However, migration inhibition of AEEA was less effective in MDA-MB-231 cells as compared to MCF 7 cells at lower concentration. On the other hand, migration of MDA-MB-231 cells was almost completely inhibited at the concentration of 400 *μ*g/mL similar to the case of MCF 7 cells (Figure 8). The % of open area (scratch) was quantified with TScratch software (ETH Zürich).

### 3.14. Acute Toxicity Study

#### 3.14.1. General Observations

In present study effect of oral administration of AEEA was observed and summarized in Table 1. Observations of this study revealed that oral administration of AEEA up to 2000 mg/kg body weight does not produce any sign of toxicity in both sexes. Body weight does not show any significant difference between control and treated groups in case of both sexes.

Biochemical and* hematological* parameters of control and treated groups were analyzed and results were summarized in Tables 2, 3, 4, 5, and 6. Results analysis suggested that the oral administration of AEEA does not produce any sign of toxicity. Biochemical parameters ALT, AST, ALP, and TBIL are the markers of hepatotoxicity, and CREA and BUN are the markers of nephrotoxicity. Analysis of these parameters shows that AEEA does not produce any adverse effect on liver and kidney function. Statistical analysis of blood parameters was carried out and results indicate no significant differences between control and treated groups. Macroscopic analysis of major vital organs was performed and the results suggested that no significant difference was observed in colour, texture, and size treated groups in comparison to control group.

#### 3.14.2. Chemical Characterization of* Eclipta alba* Extract

**Figure 9:**
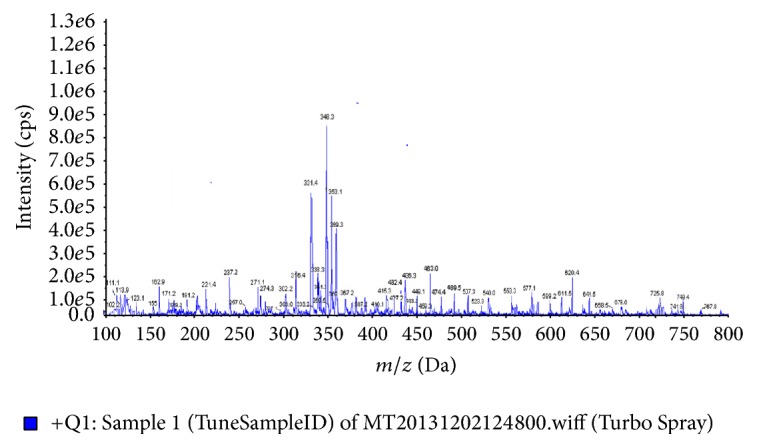
Mass fingerprinting chromatogram of SIE in positive ion (M + 1) mode.

Identification of chemical constituent of AEEA was carried out using mass spectrometry. Several compounds of AEEA were reported in various scientific literatures [345–349]. A combined analysis was done using mass spectrometric data and literature, to construct the final list of identified compounds. The result was shown in Table 7 and Figure 9.

## 4. Discussion

The role of medicinal plants as a source for medicine has been recognized since ancient time [90–92]. Although a major scientific and technological advancement has occurred in an area of combinatorial chemistry, medicinal plants form the basis for many of the drugs currently in commercial use or in development phase [93–95]. They are helping us to find out totally new chemical classes of therapeutic agents and novel mechanisms of action. Several compounds isolated from plants are currently used for the treatment of cancer and other diseases [5, 90].

*Eclipta alba* is a well-known plant of ethnomedicinal significance in the Indian subcontinent. The Ayurvedic pharmacopoeia of India considers the plant as hepatoprotective [96]. In the present study, anti-breast cancer activity of AEEA was carried out in nonmetastatic human MCF 7, metastatic human MDA-MB-231, and metastatic mouse 4T 1 cell lines and results suggested that AEEA shows concentration dependent growth inhibition in each cell line.

Apoptosis is genetically controlled programmed cell death, which takes place during the process of embryonic development, under pathological conditions, as in maintenance of tissue homeostasis, and in aging. The phenomenon of apoptosis is characterized by specific morphologic features, including loss of plasma membrane asymmetry and attachment, plasma membrane blebbing, condensation of the cytoplasm and nucleus, and internucleosomal cleavage of DNA [84].

Morphological observation was performed to detect whether the cytotoxic effect was related to apoptosis, and results suggested that treatment of breast cancer cells with AEEA causes cell shrinkage, ruffling, and blebbing of cell membrane, which are the important features of apoptosis. Fluorescent microscopic analysis of breast cancer cells treated with AEEA was carried out to observe the externalization of phosphatidylserine; a key feature of early apoptosis was observed using Annexin V FITC, which binds with phosphatidylserine and gives green fluorescence. Here, we find out that treated cells were more evident of apoptosis than control. Treatment of AEEA also results in plasma membrane disintegration, chromatin condensation, and apoptosis mediated cell death, as evident by the fluorescent microscopic analysis with propidium iodide staining. Furthermore, study of breast cancer cells stained with Hoechst 33342 was performed, and results confirmed that treatment of AEEA causes karyopyknosis and chromatin condensation in nucleus, which further results in programmed cell death. Collectively from the results of microscopy it was confirmed that AEEA induces the apoptosis mediated cell death in treated breast cancer cells.

In addition to being the source of energy that sustained the life under aerobic conditions, mitochondria can also be the source of cell signals leading to apoptosis. Several mitochondrial proteins are directly associated with this signaling event. Decline in mitochondrial membrane potential causes release of signals that initiate apoptotic cell death [97–99]. Loss in mitochondrial membrane potential was determined by using Rhodamine 123, which is a positively charged fluorescent dye. In present study loss in fluorescence was observed due to externalization of Rhodamine 123 from mitochondrial matrix and revealed that decrease in mitochondrial membrane potential occurred in AEEA treated cells dose dependently, which subsequently initiated a cascade leading to apoptosis.

In course of metastasis, cancer was spread beyond the place of origin into the other parts of body. Recurrence and metastasis of breast cancer after initial diagnosis and treatment are one of the major challenges for current therapeutic methods [100]. Prevention of metastasis is very crucial for success of breast cancer therapy and survival of patients after diagnosis and treatment. Here, we investigated the migration inhibition potential of AEEA and results suggested that it has potentially inhibited the migration of breast cancer cell lines.

Safety evaluation of AEEA was done in HEK-293 cells and CF rat to investigate the possible toxic effect of extract.* In vitro* safety evaluation in HEK-293 cells shows that it does not induce any significant toxicity. AEEA was also tested for its effects in* in vivo* system. Here no significant change in body weight, food and water intake, behaviour, or mortality was observed as compared with control group. Macroscopic parameters of vital organs also do not show any significant differences in treated groups.

In present study, serum biochemical parameters were analyzed and they do not show any statistical significant difference between control and treated groups in both sexes. Alanine aminotransferase (ALT), aspartate aminotransferase (AST), alkaline phosphatase (ALP), and total bilirubin are the liver function markers, used to evaluate the liver function. In values of liver function markers no significant change was observed among control and treated groups. Serum biochemical parameter creatinine (CRTEA), blood urea nitrogen (BUN), and UREA are used to evaluate the kidney function. Results suggested that they do not show any significant change. Triglycerides (TG), total cholesterol (TCHO), total protein (TP), albumin (ALB), total glucose (GLU), calcium (Ca), and inorganic phosphorus (IP) levels in serum were evaluated. These values represent the general metabolism of body. They also show no significant difference and dose response [101]. In conclusion, analysis of serum biochemical parameters shows that AEEA does not produce any adverse effect on liver, kidney, and general metabolic function of the body.* Hematological* parameters were observed in present study to find out the effect of AEEA on blood. No significant changes between different dose groups were observed in male as well as in female rats. Thus, AEEA oral administration does not show any adverse effect on* hematological* parameters.

Chemical analysis of AEEA was performed by mass spectrometry and result has been summarized in Table 7 and Figure 9. Table 7 consists of a list of identified compounds. Further, a literature survey on identified compounds has been performed by using citation source like Pubmed and Web of Science and result shows that a majority of identified compounds exhibit anticancer as well as antioxidant activity. Result of literature survey shows that a majority of identified compounds exhibit anticancer as well as antioxidant activity (Supporting Information: Table 1 in Supplementary Material available online at https://doi.org/10.1155/2017/9094641). Thus, we have concluded that presence of these compounds in* Eclipta alba* extract leads to its anticancer and antioxidant activity. Outcomes of chemical analysis will be helpful in further research for identification and synthesis of bioactive compounds responsible for antioxidant, anti-breast cancer, and protective activity.

At moderate concentrations reactive oxygen species (ROS) act as secondary messenger and participate in a variety of intracellular signaling pathways. An excessive and/or sustained increase in ROS production has been implicated in the pathogenesis of cancer, neurodegenerative diseases, atherosclerosis, ischemia/reperfusion injury, obstructive sleep apnea, rheumatoid arthritis, diabetes mellitus, and other diseases. Thus, free radical and ROS scavenging activity of plant extracts are very useful for protection of normal cells from oxidative damage [102]. Protective activity of AEEA for free radicals was confirmed by antioxidant activity assays. Furthermore, protective role of AEEA in cellular system was determined, and it significantly reduces the level of ROS in HEK-293 cells in concentration dependent manner. This activity is also helpful to mitigate the side effects during cancer therapy. Thus, it is to be expected that use of AEEA as an anti-breast cancer agent will not cause oxidative damage to normal cells, which are observed in case of doxorubicin, paclitaxel, and docetaxel [103].

A number of research studies in which* Eclipta alba* extract was subjected to* in vivo* treatment measured the level of glutathione (GSH), a tripeptide containing an active thiol group in the form of a cysteine residue, providing protection against reactive oxygen and nitrogen species [104]. Bhaskar et al., 2014, Mansoorali et al., 2012, Hemalakshmi et al., 2012, Arun et al., 2011, Parmar et al., 2010, and so forth described the effect of* Eclipta alba* extract treatment on level of GSH, and results of these studies reported that level of GSH was decreased during the oxidative stress. However, after that the treatment level was significantly increased. So, it was concluded that* Eclipta alba* extract helps to restore the level of GSH during oxidative stress and hence played a crucial role to provide protection against oxidative damage [105–109].

## 5. Conclusion

Our results provided a new insight into antioxidant and anti-breast cancer activity and toxicological evaluation of AEEA. Our data demonstrated that AEEA provides protection against free radical and intracellular ROS. Moreover, toxicological evaluation of AEEA provides us with crucial information which is helpful to understand its effect on* in vivo* system. The anti-breast cancer study finding demonstrated that AEEA induces apoptosis mediated cell death. These results suggested that AEEA could be a candidate for a novel anticancer agent and may have a potential to be used in complementary and alternative medicine for the treatment of breast cancer and other types of cancer.

## Supplementary Material

Result literature survey on identified compounds by using citation source like Pubmed and Web of Science.

## Figures and Tables

**Table 1:** Total phenolic and flavonoid contents and the antioxidant activity of AEEA extract along with standard ascorbic acid.

Test sample	TPC (mg/g DW)	TFC (mg/g DW)	TAA (mg/g DW)	% of inhibition at the concentration of 25 *μ*g/mL			IC_50_ (*μ*g/mL)		
				DPPH	NO	LPIA	DPPH	NO	LPIA
AEEA	31.67 ± 3.82	91.67 ± 5.05	173.33 ± 5.77	25.62	21.90	19.58	136.57 ± 6.83	308.73 ± 6.34	126.99 ± 8.10
AA			726.57 ± 9.33	63.75	47.77	36.36	33.5 ± 1.03	66.27 ± 1.55	55.64 ± 3.27

AEEA = alcoholic extract of *Eclipta alba*, AA = ascorbic acid, TPC = total phenolic contents, TFC = total flavonoid contents, TAA = total antioxidant activity, DPPH assay = 2,2-diphenyl-1-picrylhydrazyl assay, NO assay = nitric oxide assay, and LPIA = lipid peroxidation inhibition activity. Values are mean ± standard deviation.

**Table 2:** Observation for toxicity symptoms as defined in the Common Toxicity Criteria developed by the Cancer Therapy Evaluation Program (National Cancer Institute, 1999, Common Toxicity Criteria Version 2.0, Cancer Therapy Evaluation Program).

	Observation	Control group		Test groups (500 mg/kg B. Wt., 1000 mg/kg B. Wt., 1500 mg/kg B. Wt., and 2000 mg/kg B. Wt.)	
		Male	Female	Male	Female
1	Skin and fur	Normal	Normal	Normal	Normal
2	Eyes	Normal	Normal	Normal	Normal
3	Mucous membrane	Normal	Normal	Normal	Normal
4	Behavioural patterns	Normal	Normal	Normal	Normal
5	Salivation	Normal	Normal	Normal	Normal
6	Lethargy	Normal	Normal	Normal	Normal
7	Sleep	Normal	Normal	Normal	Normal
8	Diarrhea	Normal	Normal	Normal	Normal
9	Coma	NO	NO	NO	NO
10	Tremors	NO	NO	NO	NO
11	Vomiting and hematemesis (vomiting blood)	NO	NO	NO	NO
12	Hematochezia (bloody stools)	NO	NO	NO	NO
13	Loss of appetite	NO	NO	NO	NO
14	Dysphagia (inability to swallow)	NO	NO	NO	NO
15	Constipation	NO	NO	NO	NO

NO = not observed.

**Table 3:** * Hematology* results on day 15 of oral gavage administration of 500, 1000, 1500, and 2000 mg/kg B. Wt. of AEEA to male CF rats. No statistically significant differences were seen between the control group and the test group [T-RBC = total red blood cells; Hgb = haemoglobin; MCV = mean corpuscular volume; HCT = haematocrit; MCH = mean corpuscular haemoglobin; MCHC = mean corpuscular haemoglobin concentration; WBC = white blood cells; RDW = red cell distribution width; MPV = mean platelet volume; PDW = platelet distribution width; PLT = platelet].

	Parameter	Control group	500 mg/kg B. Wt.	1000 mg/kg B. Wt.	1500 mg/kg B. Wt.	2000 mg/kg B. Wt.	*p* value
1	Hgb (g/dL)	12.26 ± 0.91	12.66 ± 0.66	12.74 ± 0.46	12.74 ± 1.18	11.92 ± 0.82	NS
2	T-RBC (×10^6^/mm^3^)	6.69 ± 0.58	6.85 ± 0.22	7.06 ± 0.36	7.15 ± 0.61	7 ± 0.64	NS
3	MCV (micron^3^)	54.84 ± 3.03	54.22 ± 0.87	54.40 ± 0.4	54.74 ± 0.66	54.11 ± 1.28	NS
4	HCT (%)	36.12 ± 2.16	37.12 ± 1.06	38.42 ± 2.06	39.14 ± 3.54	37.86 ± 3.6	NS
5	MCH (pg)	18.42 ± 1.07	18.52 ± 0.73	18.04 ± 0.43	17.82 ± 0.48	17.28 ± 0.19	NS
6	MCHC (g/dL)	34 ± 1.79	34.12 ± 0.96	33.22 ± 0.81	32.54 ± 0.96	31.73 ± 1.02	NS
7	WBC × 10^3^	5.24 ± 0.53	5.21 ± 0.93	5.27 ± 0.39	5.23 ± 0.52	5.31 ± 0.36	NS
8	RDW	8.34 ± 0.39	8.66 ± 0.42	8.12 ± 0.39	8.12 ± 0.29	8.06 ± 0.47	NS
9	MPV	4.62 ± 0.61	5.32 ± 0.19	5.1 ± 0.07	5.27 ± 0.31	5.06 ± 0.5	NS
10	PDW	8.28 ± 1.22	9.10 ± 0.37	8.98 ± 0.26	9.32 ± 0.36	8.96 ± 0.46	NS
11	PLT (×10^3^/mm^3^)	603.5 ± 216.45	535 ± 103	481.8 ± 62.93	537.4 ± 161.04	489 ± 181.42	NS

NS signifies no statistical differences when the test group is compared to the control group and when the test groups are compared to each other.

**Table 4:** * Hematology* results on day 15 of oral gavage administration of 500, 1000, 1500, and 2000 mg/kg B. Wt. of AEEA to female CF rats. No statistically significant differences were seen between the control group and the test groups [T-RBC = total red blood cells; Hgb = haemoglobin; MCV = mean corpuscular volume; HCT = haematocrit; MCH = mean corpuscular haemoglobin; MCHC = mean corpuscular haemoglobin concentration; WBC = white blood cells; RDW = red cell distribution width; MPV = mean platelet volume; PDW = platelet distribution width; PLT = platelet].

	Parameter	Control group	500 mg/kg B. Wt.	1000 mg/kg B. Wt.	1500 mg/kg B. Wt.	2000 mg/kg B. Wt.	*p* value
1	Hgb (g/dL)	13.94 ± 0.44	13.62 ± 0.69	14.94 ± 0.92	14.05 ± 0.64	14.50 ± 0.53	NS
2	T-RBC (×10^6^/mm^3^)	6.9 ± 0.35	6.7 ± 0.18	8.03 ± 1.12	7.22 ± 0.28	7.79 ± 0.41	NS
3	MCV (micron^3^)	54.64 ± 1.43	55.64 ± 0.99	55.56 ± 1.13	55.33 ± 1.14	54.32 ± 0.54	NS
4	HCT (%)	37.7 ± 1.89	37.28 ± 1.47	41.8 ± 4	39.85 ± 1.21	42.3 ± 2.39	NS
5	MCH (pg)	20.16 ± 0.75	20.3 ± 0.8	19.34 ± 0.98	19.5 ± 1.3	18.64 ± 0.73	NS
6	MCHC (g/dL)	36.78 ± 0.39	36.52 ± 0.79	34.8 ± 1.94	35.23 ± 1.55	34.36 ± 1.21	NS
7	WBC × 10^3^	5.15 ± 0.39	4.85 ± 0.36	5.32 ± 0.32	4.86 ± 1.47	5.38 ± 0.54	NS
8	RDW	8.4 ± 0.37	8.06 ± 0.45	8.4 ± 0.4	8.35 ± 0.65	8.58 ± 0.45	NS
9	MPV	5.26 ± 0.09	5.28 ± 0.22	5.06 ± 0.18	5.15 ± 0.06	5.32 ± 0.19	NS
10	PDW	9.44 ± 0.67	9.62 ± 0.72	9.24 ± 0.32	9.15 ± 0.1	9.36 ± 0.27	NS
11	PLT (×10^3^/mm^3^)	387.6 ± 105.98	450.6 ± 91.73	490.2 ± 50.08	507.4 ± 55.08	519 ± 89.29	NS

NS signifies no statistical differences when the test group is compared to the control group and when the test groups are compared to each other.

**Table 5:** Result of serum biochemical analysis on day 15 of oral gavage administration of 500, 1000, 1500, and 2000 mg/kg B. Wt. of AEEA to male CF rats. NS represents the no statistical differences, when the test groups were compared to the control group.

	Parameter	Control group		500 mg/kg B. Wt.		1000 mg/kg B. Wt.		1500 mg/kg B. Wt.		2000 mg/kg B. Wt.		*p* value
		Mean	±SD	Mean	±SD	Mean	±SD	Mean	±SD	Mean	±SD	
1	UREA	22.88	3.054	25.78	1.761	25.56	2.101	27.38	5.622	26.06	2.9381	NS
2	ALT	72.94	6.697	63.56	5.792	61.02	11.654	62.04	12.858	57.22	6.4943	NS
3	AST	221.55	16.38	224.65	28.28	193.00	37.281	173.66	15.344	175.12	14.30	NS
4	ALP	964.1	532.617	1035.08	425.288	741.02	291.594	704.74	416.34	667.74	145.962	NS
5	TG	41.5	9.738	45.84	14.800	27.74	6.459	38.44	17.787	43.24	9.660	NS
6	TCHO	56.9	10.840	54.64	4.437	55.94	11.316	62.54	12.002	61.04	5.152	NS
7	TP	6.916	0.375	7.084	0.564	6.722	0.469	7.008	0.607	6.92	0.571	NS
8	ALB	3.072	0.331	2.912	0.193	3.064	0.083	3.05	0.062	2.94	0.191	NS
9	GLU	136.06	23.874	127	57.043	157.54	74.582	188.78	65.466	164.04	20.346	NS
10	Ca	9.43	1.110	9.978	0.353	9.996	0.621	10.492	0.414	9.6	0.626	NS
11	IP	9.246	1.270	8.184	0.835	8.052	1.234	8.914	0.532	7.216	0.734	NS
12	TBIL	0.17	0.065	0.154	0.03	0.134	0.032	0.178	0.070	0.112	0.028	NS
13	CREA	0.67	0.081	0.604	0.103	0.62	0.083	0.654	0.055	0.622	0.034	NS
14	BUN	10.66	1.411	12.022	0.819	11.906	0.990	12.762	2.629	12.152	1.375	NS

**Table 6:** Result of serum biochemical analysis on day 15 of oral gavage administration of 500, 1000, 1500, and 2000 mg/kg B. Wt. of AEEA to female CF rats. NS represents the no statistical differences, when the test groups were compared to the control group.

	Parameter	Control group		500 mg/kg B. Wt.		1000 mg/kg B. Wt.		1500 mg/kg B. Wt.		2000 mg/kg B. Wt.		*p* value
		Mean	±SD	Mean	±SD	Mean	±SD	Mean	±SD	Mean	±SD	
1	UREA	**30.24**	3.079	**30.38**	3.928	**33.48**	3.679	**34.96**	5.646	**30.92**	1.855	NS
2	ALT	**58.80**	17.86	**56.38**	12.41	**56.86**	5.54	**53.46**	6.72	**57.90**	5.97	NS
3	AST	**227.2**	53.05	**201.6**	43.38	**217.2**	46.69	**183.5**	29.34	**185.2**	24.32	NS
4	ALP	**660.6**	107.5	**590.4**	98.89	**608.3**	91.49	**588.5**	124.4	**675.5**	100.7	NS
5	TG	**105.3**	26.00	**104.2**	24.40	**118.8**	53.82	**134.6**	25.77	**104.5**	9.02	NS
6	TCHO	**67.00**	14.56	**65.38**	3.181	**68.70**	6.785	**67.92**	7.093	**68.60**	4.658	NS
7	TP	**7.08**	0.356	**6.914**	0.622	**7.480**	0.493	**7.664**	0.444	**7.240**	0.327	NS
8	ALB	**3.360**	0.103	**3.354**	0.190	**3.520**	0.205	**3.550**	0.232	**3.508**	0.124	NS
9	GLU	**102.4**	26.65	**123.4**	28.76	**123.20**	31.75	**132.90**	58.95	**128.80**	46.80	NS
10	Ca	**9.060**	0.468	**8.783**	0.345	**9.348**	0.910	**9.928**	0.749	**9.726**	0.352	NS
11	IP	**10.52**	0.540	**10.46**	1.645	**11.34**	3.065	**11.39**	1.564	**11.38**	1.477	NS
12	TBIL	**0.075**	0.001	**0.078**	0.026	**0.077**	0.022	**0.068**	0.023	**0.074**	0.011	NS
13	CREA	**0.682**	0.076	**0.70**	0.078	**0.712**	0.150	**0.774**	0.040	**0.672**	0.037	NS
14	BUN	**14.09**	1.432	**14.16**	1.825	**15.61**	1.698	**16.29**	2.64	**14.42**	0.874	NS

**Table 7:** Summary of compounds found in extract of AEEA.

Compounds of *Eclipta alba* extract		
Sl number	Compounds	Mol. Wt.
1	2-Methylbutanal oxime	101
2	Catechol	110
3	Uracil	112
4	Phenyl ethylamine	122
5	Nicotinic acid	123.12
6	4-Hydroxybenzoic acid	138.13
7	3,4-Dihydroxybenzoic acid	154.13
8	Dihydrocarveol	154.28
9	L-Nicotine	162.26
10	Gallic acid	170
11	Catechol derivative	190
12	Caryophyllene oxide	220.39
13	Coumestan	236.23
14	Apigenin	270.25
15	Butein	272.27
16	*α*-Terthienyl methanol	278.44
17	Indolylmethyl glucosinolate	283
18	Luteolin	286.25
19	Testosterone	288.47
20	2-Terthiophene-5-carboxylic acid	292.42
21	Demethylwedelolactone	302.25
22	Wedelolactone	315.1
23	Tyramine *β* xanthine	330
24	Gallic acid hexoxide	332
25	Quercetin derivative	347
26	Catechin derivative	352
27	Quercetin derivative	358
28	16-Methoxytabersonine	367
29	Stigmasterol	384.71
30	*β*-Sitosterol	414
31	*β*-Amyrin	426.8
32	Hypophyllanthin	430
33	Apigenin-7-O-glucoside	432.41
34	Epicatechin	442
35	Quercetin-3-rhamnoside	448
36	Cynaroside	448.41
37	Catechin derivative	458
38	Demethylwedelolactone 7-glucoside	462.39
39	Galloyl-isorhamnetin	468
40	Echinocystic acid	472.78
41	Caulophyllogenin	488.78
42	Myoinositol	492
