# Supplementary material for: Alcoholic Extract of Eclipta alba Shows In Vitro Antioxidant and Anticancer Activity without Exhibiting Toxicological Effects
Source: Oxid Med Cell Longev. 2017 Jan 31;2017:9094641. doi: 10.1155/2017/9094641 (PMC5307245; doi:10.1155/2017/9094641)
Supplement: Supplementary file 1 — Result literature survey on identified compounds by using citation source like Pubmed and Web of Science. [file 9094641.f1.docx]

| **S.No.** | **Compounds** | **Mol. Wt** | **Anticancer activity (AC)** | **Antioxidant activity (AO)** | **References** |
| --- | --- | --- | --- | --- | --- |
| 1. 1 | 2,methylbutanal oxime | 101 | NA | NA |  |
| 1. 2 | Catechol | 110 | YES | YES | [[1](#_ENREF_1),[2](#_ENREF_2)] AO |
| 1. 3 | Uracil | 112 | YES | YES | [[3](#_ENREF_3)] AC [[4](#_ENREF_4)] AO |
| 1. 4 | Phenyl ethylamine | 122 | NA | NA |  |
| 1. 5 | Nicotinic acid | 123.12 | YES | YES | [[5](#_ENREF_5)] BOTH |
| 1. 6 | 4-hydroxybenzoic acid | 138.13 | YES | YES | [[6](#_ENREF_6)]BOTH |
| 1. 7 | 3,4-dihydroxybenzoic acid | 154.13 | YES | YES | [[7](#_ENREF_7)] BOTH |
| 1. 8 | Dihydrocarveol | 154.28 | NA | NA |  |
| 1. 9 | L-nicotene | 162.26 | NA | NA |  |
| 1. 10 | Gallic acid | 170 | YES | YES | [[8](#_ENREF_8)],AO  [[9](#_ENREF_9)] AC |
| 1. 11 | Catechol derivative | 190 | YES | YES | [[1](#_ENREF_1)]AC, [[10](#_ENREF_10)]AO |
| 1. 12 | Caryophyllene oxide | 220.39 | NA | NA |  |
| 1. 13 | Coumestan | 236.23 | YES | YES | [[11](#_ENREF_11)]AC, [[12](#_ENREF_12)] AO |
| 1. 14 | Apigenin | 270.25 | YES | YES | [[13](#_ENREF_13)]AO, [[14](#_ENREF_14)] AC, [[15](#_ENREF_15)] AC |
| 1. 15 | Butein | 272.27 | YES | YES | [[16](#_ENREF_16)]AO, [[17](#_ENREF_17)] AC |
| 1. 16 | α-terthienyl methanol | 278.44 | YES | NA | [[18](#_ENREF_18)] AC |
| 1. 17 | Indolylmethyl glucosinolate | 283 | NA | NA |  |
| 1. 18 | Luteolin | 286.25 | YES | YES | [[19](#_ENREF_19)]AO [[20](#_ENREF_20)] AO, [[21](#_ENREF_21)] AC |
| 1. 19 | Testosterone | 288.47 | NA | NA |  |
| 1. 20 | 2-Terthiophene-5-carboxylic acid | 292.42 | NA | NA |  |
| 1. 21 | Demethylwedelolactone | 302.25 | YES | YES | [[22](#_ENREF_22)]AC, [[23](#_ENREF_23)] AO |
| 1. 22 | Wedelolactone | 315.1 | YES | YES | [[22](#_ENREF_22)] AC, [[24](#_ENREF_24)] AO |
| 1. 23 | Tyramine β xanthine | 330 | NA | NA |  |
| 1. 24 | Gallic acid hexoxide | 332 | NA | NA |  |
| 1. 25 | Quercetin derivative | 347,358 | YES | YES | [[25](#_ENREF_25)] AC, [[26](#_ENREF_26)]AO |
| 1. 26 | Catechin derivative | 352,458 | YES | YES | [[27](#_ENREF_27)]AO, [[28](#_ENREF_28)]AO, [[29](#_ENREF_29)] AC |
| 1. 28 | 16methoxytabersonine | 367 | NA | NA |  |
| 1. 29 | Stigmasterol | 384.71 | YES | YES | [[30](#_ENREF_30)]AC, [[31](#_ENREF_31)]AO, |
| 1. 30 | β sitosterol | 414 | YES | YES | [[32](#_ENREF_32)]AO, [[33](#_ENREF_33)]AO, [[34](#_ENREF_34)]AC |
| 1. 31 | β-amyrin | 426.8 | NA | YES | [[35](#_ENREF_35)]AO |
| 1. 32 | Hypophyllanthin | 430 | YES | YES | [[36](#_ENREF_36)]AC, [[37](#_ENREF_37)] AC, [[38](#_ENREF_38)]AO, [[39](#_ENREF_39)]AO |
| 1. 33 | Apigenin-7-O-glucoside | 432.41 | YES | YES | [[15](#_ENREF_15)] AC [[40](#_ENREF_40)] AO |
| 1. 34 | Epicatechin | 442 | YES | YES | [[41](#_ENREF_41)]AO, [[42](#_ENREF_42)]AC, [[43](#_ENREF_43)]AC, [[44](#_ENREF_44)]AC |
| 1. 35 | Quercetin-3-rhamnoside | 448 | YES | YES | [[45](#_ENREF_45)] AO, [[46](#_ENREF_46)] AC & AO |
| 1. 36 | Cynaroside | 448.41 | YES | YES | [[47](#_ENREF_47)]AO, [[48](#_ENREF_48)]AC & AO, [[49](#_ENREF_49)] AO |
| 1. 38 | Demethylwedelolactone 7-glucoside | 462.39 | YES | YES | [[50](#_ENREF_50)] AC, [[51](#_ENREF_51)] AO. |
| 1. 39 | Galloyl-isorhamnetin | 468 | NA | NA |  |
| 1. 40 | Echinocystic acid | 472.78 | YES | NA | [[52](#_ENREF_52)]AC, |
| 1. 41 | Caulophyllogenin | 488.78 | NA | NA |  |
| 1. 42 | Myoinositol | 492 | YES | YES | [[53](#_ENREF_53)] AC, [[54](#_ENREF_54)] AC, [[55](#_ENREF_55)] AO |

NA not applicable

**Reference**

1. Nair PR, Melnick SJ, Wnuk SF, Rapp M, Escalon E, et al. (2009) Isolation and characterization of an anticancer catechol compound from Semecarpus anacardium. Journal of ethnopharmacology 122: 450-456.

2. Kumar S, Pandey AK (2013) Chemistry and biological activities of flavonoids: an overview. The Scientific World Journal 2013.

3. Hassib ST (1981) Qualitative and quantitative analysis of uracil anticancer drugs. Talanta 28: 685-687.

4. Akhatova G, Safarova I, Gerchikov AY (2011) Antioxidant activity of uracil derivatives. Kinetics and Catalysis 52: 1-5.

5. Perumal SS, Shanthi P, Sachdanandam P (2005) Augmented efficacy of tamoxifen in rat breast tumorigenesis when gavaged along with riboflavin, niacin, and CoQ 10: effects on lipid peroxidation and antioxidants in mitochondria. Chemico-biological interactions 152: 49-58.

6. Spilioti E, Jaakkola M, Tolonen T, Lipponen M, Virtanen V, et al. (2014) Phenolic acid composition, antiatherogenic and anticancer potential of honeys derived from various regions in Greece. PloS one 9: e94860.

7. Lin HH, Chen JH, Huang CC, Wang CJ (2007) Apoptotic effect of 3, 4‐dihydroxybenzoic acid on human gastric carcinoma cells involving JNK/p38 MAPK signaling activation. International Journal of Cancer 120: 2306-2316.

8. Abdelwahed A, Bouhlel I, Skandrani I, Valenti K, Kadri M, et al. (2007) Study of antimutagenic and antioxidant activities of Gallic acid and 1, 2, 3, 4, 6-pentagalloylglucose from Pistacia lentiscus: Confirmation by microarray expression profiling. Chemico-biological interactions 165: 1-13.

9. Chen H-M, Wu Y-C, Chia Y-C, Chang F-R, Hsu H-K, et al. (2009) Gallic acid, a major component of Toona sinensis leaf extracts, contains a ROS-mediated anti-cancer activity in human prostate cancer cells. Cancer letters 286: 161-171.

10. Miura T, Muraoka S, Ogiso T (1998) Antioxidant activity of adrenergic agents derived from catechol. Biochemical pharmacology 55: 2001-2006.

11. Nehybova T, Smarda J, Benes P (2014) Plant coumestans: recent advances and future perspectives in cancer therapy. Anti-Cancer Agents in Medicinal Chemistry (Formerly Current Medicinal Chemistry-Anti-Cancer Agents) 14: 1351-1362.

12. Xi G-L, Liu Z-Q (2014) Coumestan inhibits radical-induced oxidation of DNA: is hydroxyl a necessary functional group? Journal of agricultural and food chemistry 62: 5636-5642.

13. Romanova D, Vachalkova A, Cipak L, Ovesna Z, Rauko P (2000) Study of antioxidant effect of apigenin, luteolin and quercetin by DNA protective method. Neoplasma 48: 104-107.

14. Ruela-de-Sousa R, Fuhler G, Blom N, Ferreira C, Aoyama H, et al. (2010) Cytotoxicity of apigenin on leukemia cell lines: implications for prevention and therapy. Cell death & disease 1: e19.

15. Shukla S, Gupta S (2010) Apigenin: a promising molecule for cancer prevention. Pharmaceutical research 27: 962-978.

16. Cheng Z-J, Kuo S-C, Chan S-C, Ko F-N, Teng C-M (1998) Antioxidant properties of butein isolated from Dalbergia odorifera. Biochimica et Biophysica Acta (BBA)-Lipids and Lipid Metabolism 1392: 291-299.

17. Zhang L, Chen W, Li X (2008) A novel anticancer effect of butein: Inhibition of invasion through the ERK1/2 and NF‐κB signaling pathways in bladder cancer cells. FEBS letters 582: 1821-1828.

18. Lee J-S, Ahn J-H, Cho Y-J, Kim H-Y, Yang Y-I, et al. (2015) α-Terthienylmethanol, isolated from Eclipta prostrata, induces apoptosis by generating reactive oxygen species via NADPH oxidase in human endometrial cancer cells. Journal of ethnopharmacology 169: 426-434.

19. Sato Y, Sasaki N, Saito M, Endo N, Kugawa F, et al. (2015) Luteolin attenuates Doxorubicin-induced cytotoxicity to mcf-7 human breast cancer cells. Biological and Pharmaceutical Bulletin 38: 703-709.

20. Huang C-S, Lii C-K, Lin A-H, Yeh Y-W, Yao H-T, et al. (2013) Protection by chrysin, apigenin, and luteolin against oxidative stress is mediated by the Nrf2-dependent up-regulation of heme oxygenase 1 and glutamate cysteine ligase in rat primary hepatocytes. Archives of toxicology 87: 167-178.

21. Arya RK, Singh A, Yadav NK, Cheruvu SH, Hossain Z, et al. (2015) Anti-breast tumor activity of Eclipta extract in-vitro and in-vivo: novel evidence of endoplasmic reticulum specific localization of Hsp60 during apoptosis. Scientific reports 5.

22. Lee Y-J, Lin W-L, Chen N-F, Chuang S-K, Tseng T-H (2012) Demethylwedelolactone derivatives inhibit invasive growth in vitro and lung metastasis of MDA-MB-231 breast cancer cells in nude mice. European journal of medicinal chemistry 56: 361-367.

23. Jadhav V, Thorat R, Kadam V, Salaskar K (2009) Chemical composition, pharmacological activities of Eclipta alba. Journal of Pharmacy Research 2: 1229-1231.

24. Karthikumar S, Vigneswari K, Jegatheesan K (2007) Screening of antibacterial and antioxidant activities of leaves of Eclipta prostrata (L). Sci Res Essays 2: 101-104.

25. Hirpara KV, Aggarwal P, Mukherjee AJ, Joshi N, Burman AC (2009) Quercetin and its derivatives: synthesis, pharmacological uses with special emphasis on anti-tumor properties and prodrug with enhanced bio-availability. Anti-Cancer Agents in Medicinal Chemistry (Formerly Current Medicinal Chemistry-Anti-Cancer Agents) 9: 138-161.

26. Kessler M, Ubeaud G, Jung L (2003) Anti‐and pro‐oxidant activity of rutin and quercetin derivatives. Journal of Pharmacy and Pharmacology 55: 131-142.

27. Nanjo F, Goto K, Seto R, Suzuki M, Sakai M, et al. (1996) Scavenging effects of tea catechins and their derivatives on 1, 1-diphenyl-2-picrylhydrazyl radical. Free Radical Biology and Medicine 21: 895-902.

28. Fukuhara K, Nakanishi I, Ohkubo K, Obara Y, Tada A, et al. (2009) Intramolecular base-accelerated radical-scavenging reaction of a planar catechin derivative bearing a lysine moiety. Chemical Communications: 6180-6182.

29. Kumar D, Harshavardhan S, Chirumarry S, Poornachandra Y, Jang K, et al. (2015) Design, Synthesis In Vitro Anticancer Activity and Docking Studies of (−)‐Catechin Derivatives. Bulletin of the Korean Chemical Society 36: 564-570.

30. Bradford PG, Awad AB (2007) Phytosterols as anticancer compounds. Molecular nutrition & food research 51: 161-170.

31. Yoshida Y, Niki E (2003) Antioxidant effects of phytosterol and its components. Journal of nutritional science and vitaminology 49: 277-280.

32. Baskar AA, Al Numair KS, Gabriel Paulraj M, Alsaif MA, Muamar MA, et al. (2012) β-sitosterol prevents lipid peroxidation and improves antioxidant status and histoarchitecture in rats with 1, 2-dimethylhydrazine-induced colon cancer. Journal of medicinal food 15: 335-343.

33. Gupta R, Sharma AK, Dobhal M, Sharma M, Gupta R (2011) Antidiabetic and antioxidant potential of β‐sitosterol in streptozotocin‐induced experimental hyperglycemia. Journal of diabetes 3: 29-37.

34. Baskar AA, Ignacimuthu S, Paulraj GM, Al Numair KS (2010) Chemopreventive potential of β-sitosterol in experimental colon cancer model-an in vitro and in vivo study. BMC complementary and alternative medicine 10: 1.

35. Sunil C, Irudayaraj SS, Duraipandiyan V, Al-Dhabi NA, Agastian P, et al. (2014) Antioxidant and free radical scavenging effects of β-amyrin isolated from S. cochinchinensis Moore. leaves. Industrial Crops and Products 61: 510-516.

36. Islam A, Selvan T, Mazumder U, Gupta M, Ghosal S (2008) Antitumour effect of phyllanthin and hypophyllanthin from Phyllanthus amarus against Ehrlich ascites carcinoma in mice. Pharmacologyonline 2: 796-807.

37. Parvathaneni M, Battu GR, Gray AI, Gummalla P (2014) Investigation of anticancer potential of hypophyllanthin and phyllanthin against breast cancer by in vitro and in vivo methods. Asian Pacific Journal of Tropical Disease 4: S71-S76.

38. Kandhare AD, Ghosh P, Ghule AE, Zambare GN, Bodhankar SL (2013) Protective effect of Phyllanthus amarus by modulation of endogenous biomarkers and DNA damage in acetic acid induced ulcerative colitis: Role of phyllanthin and hypophyllanthin. Apollo Medicine 10: 87-97.

39. Thippeswamy A, Shirodkar A, Koti B, Sadiq AJ, Praveen D, et al. (2011) Protective role of Phyllantus niruri extract in doxorubicin-induced myocardial toxicity in rats. Indian journal of pharmacology 43: 31.

40. Burda S, Oleszek W (2001) Antioxidant and antiradical activities of flavonoids. Journal of agricultural and food chemistry 49: 2774-2779.

41. Iacopini P, Baldi M, Storchi P, Sebastiani L (2008) Catechin, epicatechin, quercetin, rutin and resveratrol in red grape: Content, in vitro antioxidant activity and interactions. Journal of Food Composition and Analysis 21: 589-598.

42. Rodriguez M, Du G-J, Wang C-Z, Yuan C-S (2010) Letter to the editor: Panaxadiol's anticancer activity is enhanced by epicatechin. The American journal of Chinese medicine 38: 1233-1235.

43. Rodgers EH, Grant MH (1998) The effect of the flavonoids, quercetin, myricetin and epicatechin on the growth and enzyme activities of MCF7 human breast cancer cells. Chemico-biological interactions 116: 213-228.

44. Ravindranath MH, Saravanan TS, Monteclaro CC, Presser N, Ye X, et al. (2006) Epicatechins purified from green tea (Camellia sinensis) differentially suppress growth of gender-dependent human cancer cell lines. Evidence-Based Complementary and Alternative Medicine 3: 237-247.

45. Singh AP, Wilson T, Kalk AJ, Cheong J, Vorsa N (2009) Isolation of specific cranberry flavonoids for biological activity assessment. Food chemistry 116: 963-968.

46. Azab SS, Abdel-Daim M, Eldahshan OA (2013) Phytochemical, cytotoxic, hepatoprotective and antioxidant properties of Delonix regia leaves extract. Medicinal Chemistry Research 22: 4269-4277.

47. Sun X, Sun Gb, Wang M, Xiao J, Sun Xb (2011) Protective effects of cynaroside against H2O2‐induced apoptosis in H9c2 cardiomyoblasts. Journal of cellular biochemistry 112: 2019-2029.

48. Mamadalieva NZ, Herrmann F, El‐Readi MZ, Tahrani A, Hamoud R, et al. (2011) Flavonoids in Scutellaria immaculata and S. ramosissima (Lamiaceae) and their biological activity. Journal of Pharmacy and Pharmacology 63: 1346-1357.

49. Žemlička L, Fodran P, Lukeš V, Vagánek A, Slováková M, et al. (2014) Physicochemical and biological properties of luteolin-7-O-β-d-glucoside (cynaroside) isolated from Anthriscus sylvestris (L.) Hoffm. Monatshefte für Chemie-Chemical Monthly 145: 1307-1318.

50. Mithun N, Shashidhara S, Vivek Kumar R (2011) Eclipta alba (L.) A review on its phytochemical and pharmacological profile. Pharmacologyonline 1: 345-357.

51. Chokotia LS, Vashistha P, Sironiya R, Matoli H (2013) Pharmacological Activities of Eclipta Alba (L.).

52. Tong X, Lin S, Fujii M, Hou D-X (2004) Molecular mechanisms of echinocystic acid-induced apoptosis in HepG2 cells. Biochemical and biophysical research communications 321: 539-546.

53. Vucenik I, Shamsuddin AM (2006) Protection against cancer by dietary IP6 and inositol. Nutrition and cancer 55: 109-125.

54. Lee H-J, Lee S-A, Choi H (2005) Dietary administration of inositol and/or inositol-6-phosphate prevents chemically-induced rat hepatocarcinogenesis. Asian Pac J Cancer Prev 6: 41-47.

55. Jiang W-D, Wu P, Kuang S-Y, Liu Y, Jiang J, et al. (2011) Myo-inositol prevents copper-induced oxidative damage and changes in antioxidant capacity in various organs and the enterocytes of juvenile Jian carp (Cyprinus carpio var. Jian). Aquatic toxicology 105: 543-551.
